# Epigenetic Silencing of the Key Antioxidant Enzyme Catalase in Karyotypically Abnormal Human Pluripotent Stem Cells

**DOI:** 10.1038/srep22190

**Published:** 2016-02-25

**Authors:** Mikko Konki, Kalyan Pasumarthy, Maia Malonzo, Annele Sainio, Cristina Valensisi, Mirva Söderström, Maheswara Reddy Emani, Aki Stubb, Elisa Närvä, Bishwa Ghimire, Asta Laiho, Hannu Järveläinen, Riitta Lahesmaa, Harri Lähdesmäki, R. David Hawkins, Riikka J. Lund

**Affiliations:** 1 Turku Centre for Biotechnology, University of Turku and Åbo Akademi University, Turku, Finland; 2 Department of Information and Computer Science, Aalto University School of Science, Helsinki, Finland; 3 Department of Medical Biochemistry and Genetics, University of Turku, and Department of Medicine, Division of Endocrinology, Turku University Hospital, Turku, Finland; 4 Department of Medicine, Division of Medical Genetics and Department of Genome Sciences, University of Washington School of Medicine, Seattle, WA, USA; 5 Department of Pathology, Turku University Hospital, University of Turku, Turku, Finland; 6 Centre for Stem Cell Biology, the Department of Biomedical Science, University of Sheffield, Sheffield, UK

## Abstract

Epigenomic regulation is likely to be important in the maintenance of genomic integrity of human pluripotent stem cells, however, the mechanisms are unknown. We explored the epigenomes and transcriptomes of human pluripotent stem cells before and after spontaneous transformation to abnormal karyotypes and in correlation to cancer cells. Our results reveal epigenetic silencing of Catalase, a key regulator of oxidative stress and DNA damage control in abnormal cells. Our findings provide novel insight into the mechanisms associated with spontaneous transformation of human pluripotent stem cells towards malignant fate. The same mechanisms may control the genomic stability of cells in somatic tissues.

Human pluripotent stem cells (hPSC) are valuable tool for regenerative medicine, disease modelling and drug research. However, genomic abnormalities frequently accumulate in hPSC lines during *in vitro* maintenance^1^,^2^. In addition, transcriptome changes^3^,^4^,^5^ and epigenetic instability of chromosome X, imprinted and developmental genes has been observed through targeted analysis^1^,^6^. Yet the cause for these abnormalities remains unknown. Epigenetic mechanisms are likely to be important in the maintenance of genomic integrity, however, detailed studies are still lacking and no consistent epigenetic alterations have been reported in hPSCs^1^. The abnormalities accumulating in hPSCs may compromise their quality and suitability for the downstream applications by altering growth, differentiation and malignant potential of the cells. Elucidation of such alterations is, therefore, important and is expected to reveal novel insights into the mechanisms how stem cells maintain or loose the genomic balance. The same mechanisms may also have relevance for the renewal of tissues or development of malignant growth in somatic tissues.

In this study we have examined whether loss of genomic stability in hPSCs is associated with common epigenetic alterations across karyotypically abnormal hPSC lines, whether these changes affect transcriptional regulation, and if there is correlation with human cancers.

## Results and Discussion

**Figure 1:**
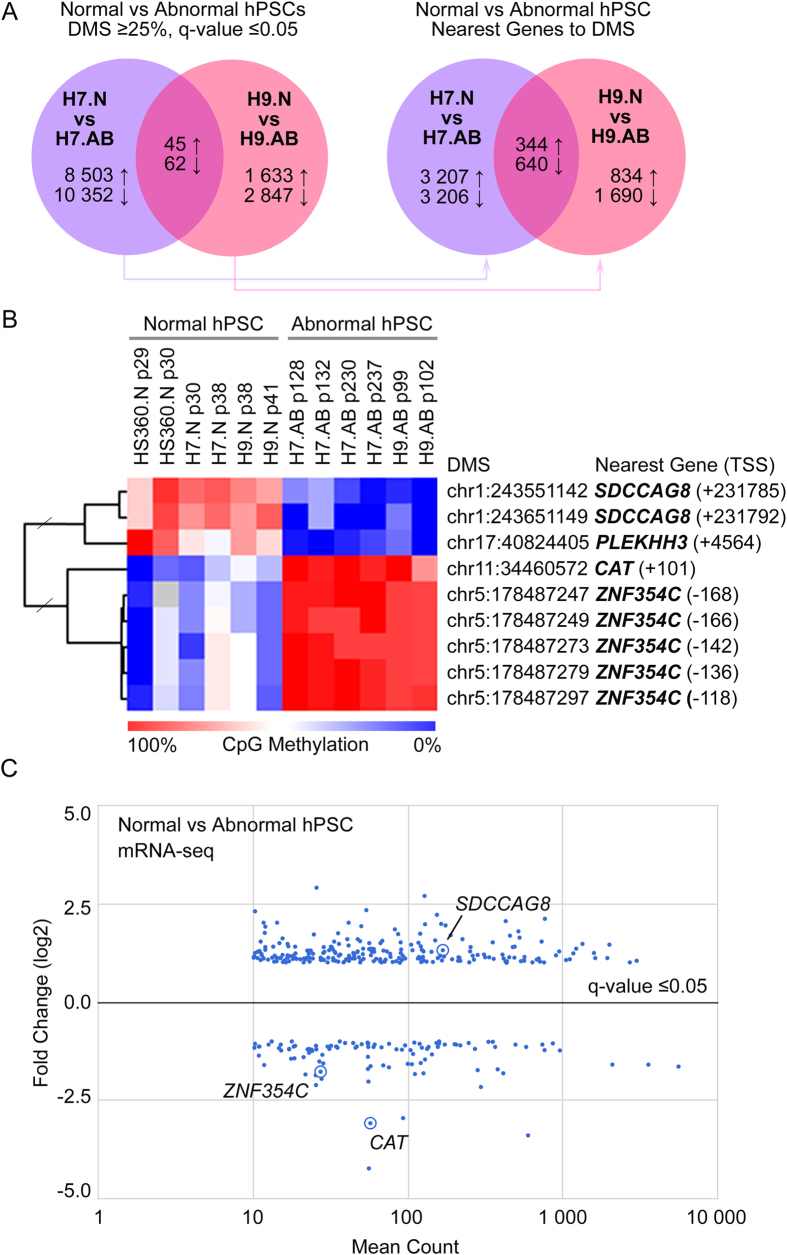
DNA Methylome and Gene Expression Differences in Karyotypically Abnormal and Normal Human Pluripotent Stem Cells. The DNA methylomes of karyotypically normal (N) or abnormal (AB) human Pluripotent Stem Cells (hPSC) were analyzed with Reduced Representation Bisulfite Sequencing. (**A**) In the left panel is the number of individual Differentially Methylated Sites (DMS) in karyotypically abnormal (H7, H9) hPSC lines when compared to normal lines (H7, H9) with tendency to accumulate karyotypic abnormalities (↑ = increased, ↓ = decreased methylation). In the right panel are the corresponding numbers of nearest genes (5 kb upstream, 1 kb downstream and max 50 kb extension) to the DMSs indicated in Fig. 1A and their overlap in H7 and H9 lines. (**B**) The CpG sites with minimum of 25% methylation difference between normal and abnormal hPSCs throughout the lines, including HS360 with stable karyotype. The nearest genes and their transcription start sites within closest distance to differentially methylated sites are indicated in the figure. (**C**) Transcriptome differences (fold change ≥2, q-value ≤0.05) between karyotypically normal and abnormal hPSCs as measured with mRNA-sequencing. The genes overlapping with the DNA methylome data (Fig. 1B) are highlighted in the figure. See Supplementary Table SI,II for numeric data.

To examine altered regulation of gene activity in hPSCs before and after spontaneous transformation to abnormal karyotype we carried out integrative epigenomic and transcriptomic analysis. In order to profile the epigenetic signatures, we analysed the CpG rich regions of the genome with single nucleotide resolution by using Reduced Representation Bisulfite Sequencing (RRBS)^7^,^8^. The investigated cell lines included hESC lines, which maintain stable karyotype (HS360) in culture as well as hESC lines (H7 and H9) with tendencies to accumulate abnormalities. Comparisons of the normal to respective abnormal hESC lines revealed 18 855 differentially methylated individual CpG sites (DMS) in H7 line and 4 480 in H9 lines (q-value ≤0.05, average methylation difference ≥25%). The nearest genes to these sites (5 kb upstream, 1 kb downstream and max 50 kb extension) included 98*4* overlapping genes in both lines (Fig. 1A, Table SI). Of these genes 23 also displayed alterations in gene expression with fold change ≥2.0 and adj.p-value <0.05. Pathway analysis revealed enrichment of the altered genes to top functional categories regulating pluripotency, cytoskeleton, cell adhesion, development and cancer (Fig. S1).

Next we examined at the single nucleotide resolution which of the individual DMS overlap between normal and abnormal cells in both H7 and H9 lines and show at least 25% methylation difference between each replicated comparison. This revealed that only 11 CpG sites were common and differentially methylated in a consistent manner. When we included in the analysis HS360 line, which does not tend to accumulate genomic abnormalities in culture, we found common methylation change in abnormal cells throughout the lines in only nine sites with minimum methylation difference of 25% (Fig. 1B, Table SII). The genes within closest distance to these sites included four genes: regulator of oxidative stress response Catalase (*CAT*), serologically defined colon cancer antigen 8 (*SDCCAG8*) and two genes with less well known function, pleckstrin homology domain containing, family H (with MyTH4 domain) member 3 (*PLEKHH3)* and zinc finger protein 354C (*ZNF354C)* (Fig. 1B). Of these, three displayed clear changes in gene expression (Fig. 1C, Table SII).

**Figure 2:**
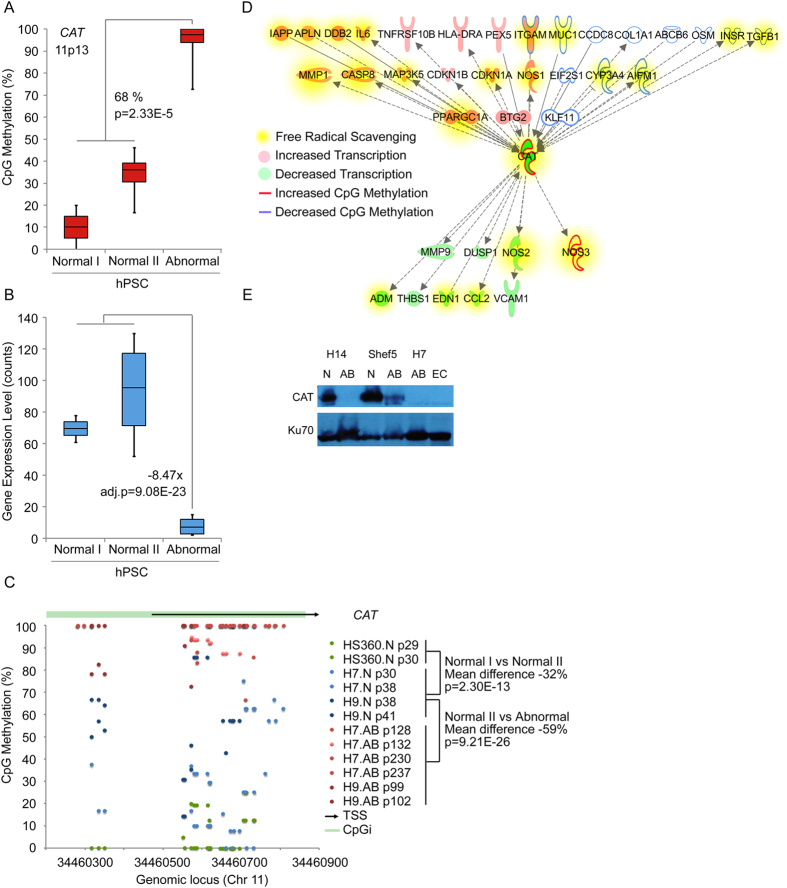
Epigenetic silencing of A Key Antioxidant Enzyme Catalase in Karyotypically Abnormal Human Pluripotent Stem Cells. (**A**) DNA methylation levels of the differentially methylated single CpG site in Catalase (*CAT*) gene in karyotypically normal cells with stable karyotype (normal I), normal cells with tendency to accumulate abnormalities (normal II) and karyotypically abnormal (abnormal) hPSCs. (**B**) Gene expression levels of *CAT* gene in karyotypically normal I, normal II and abnormal hPSCs. (**C**) DNA methylation levels of the CpG island in the Catalase (*CAT*) gene in karyotypically normal cells which maintain stable karyotype (green), which display tendency to accumulate abnormalities (blue) and karyotypically abnormal hPSCs (red). (**D**) *CAT* interacting molecules, which are differentially expressed (fold change ≥1.5, p ≤ 0.05) and/or nearest to the differentially methylated sites between karyotypically normal and abnormal H7 and H9 hPSCs (average methylation difference ≥25%). Molecules implicated in free radical scavenging (n = 23, p < 1E-9) are highlighted in the figure. (**E**) Western blot analysis of CAT protein levels in karyotypically normal (N) or abnormal (AB) cells and in NT2D1 embryonal carcinoma (EC) cells. Ku70 is a loading control. See Table SII for numeric data.

Catalase (*CAT*) stood out from the other genes due to the strongest alterations in both epigenetic (average difference 68%, p = 2.33E-5) and transcriptional regulation (fold change −8.47 adj.p = 9.08E-23) (Fig. 2A,B, Table SII). The common DMS in the *CAT* promoter was localized in a single CpG site 101 base pairs downstream of the transcription start site. Closer manual examination of the promoter area revealed differential methylation of several sites in normal and abnormal cells, although not all of them were captured by the MethylKit statistical algorithm in each RRBS dataset. The methylation level in CAT overlapping CpG increased gradually from lines not displaying tendency for alteration to those prone to alterations (Normal I vs Normal II: mean methylation difference −32%, p = 2.30E-13) and was fully methylated in the abnormal lines (Normal II vs Abnormal: mean methylation difference −59%, p = 9.21E-23, Fig. 2C). The differentially methylated CpG overlapped also with several transcription factor binding sites indicating important regulatory function^9^,^10^,^11^.

Catalase is a key mediator of responses protecting cells against oxidative stress and DNA damage. In order to get more extensive view of the signalling events potentially linked to silencing of *CAT* in abnormal hPSCs, we utilized Ingenuity Pathway Analysis Tool (Qiagen) to retrieve all the molecules known to interact with CAT. Then we examined the transcriptional (data from Table SIg) and epigenetic (data from Table SIf) regulation of this network in karyotypically normal vs abnormal cells. HS360 was excluded from the methylation data to relax the analysis parameters. The key role of *CAT* in the abnormal cells was further supported by this network analysis which revealed that several molecules (n = 36 of 147) known to interact with *CAT* were showing changes in the gene expression or were localized closest to differentially methylated CpG sites (Fig. 2D). According to Ingenuity Pathway Analysis Tool (Fisher’s exact test), these molecules were functionally associated to cell death and survival (n = 35, p = 1.99E-23–6.17E-2), development, cell growth and proliferation (n = 33, p = 1.21E-20–4.61E-2) and importantly to free radical scavenging (n = 23, p = 3.56E-18–2.25E-10).

In order to explore whether the hypermethylation and loss of transcription had led to loss of CAT protein, we carried out western blot analysis in additional pairs of normal and abnormal hPSC lines. All abnormal hPSC lines as well as embryonal carcinoma line revealed loss of CAT protein in comparison to karyotypically normal hPSCs (Fig. 2E).

**Figure 3:**
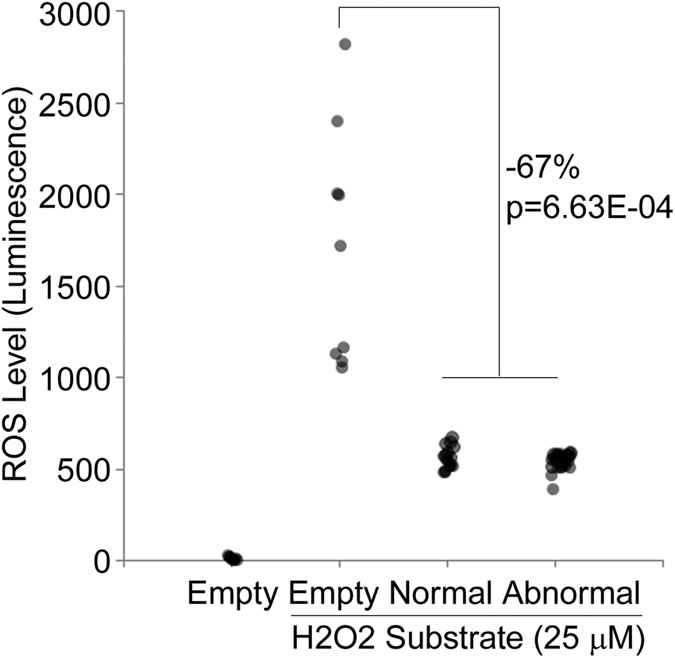
Karyotypically Normal and Abnormal Pluripotent Stem Cells Have Similar Capacity to Eliminate Reactive Oxygen Species. Human PSC lines were plated in the density of 5 000 cells per 96-well on Matrigel-coated wells in 100 ul mTeSR1 medium. Control wells without cells were included in the analysis. In order to measure the Reactive Oxygen Species responses hydrogen peroxide (H_2_O_2_) substrate (25 μM) was added into the indicated conditions for 2 hours after which the ROS-Glo detection reagent was added. The luminescence signal, proportional to H_2_O_2_ concentration, was measured from the cell culture wells in the absence (Empty) or presence of hPSC lines with normal or abnormal karyotype (trisomy of chr 12) as indicated. In the figure is representative data from the replicated measurements. The average decrease in the luminescence signal between wells without and with cells with t-test p-value is indicated in the figure.

The CAT enzyme is known to have a key function in converting reactive oxygen species (ROS), hydrogen peroxide (H_2_O_2_), to water and oxygen and thereby defending cells against oxidative stress. Thus, we next compared the capacity of karyotypically normal and abnormal cells to eliminate H_2_O_2_. Interestingly, we observed that the levels of H_2_O_2_ were high in the standard hPSC culture conditions, in the absence of cells. This indicates that H_2_O_2_ was generated abiotically in the absence of cells in the matrigel-coated wells with mTeSR1 culture media. In the presence of hESCs the H_2_O_2_ levels were decreased in average 67% (p < 0.005) indicating that the cells were able to eliminate a large proportion of the H_2_O_2_ present in the culture medium (Fig. 3). Interestingly, we did not observe any differences between the karyotypically normal and abnormal cells in the capacity to eliminate H_2_O_2_ in the culture medium. This suggests that decomposition of H_2_O_2_ by hESCs is not dependent of CAT and may be compensated by the other components of the Free Radical Scavenging Pathway some of which show changes in expression (Fig. 2D). However, silencing of *CAT* was the most strongest and consistent alteration we observed in karyotypically abnormal hESCs indicating that it potentially has a functional significance in the transformation to abnormal phenotype. Further studies are required to demonstrate the detailed mechanisms and functional significance of CAT in hESCs.

**Figure 4:**
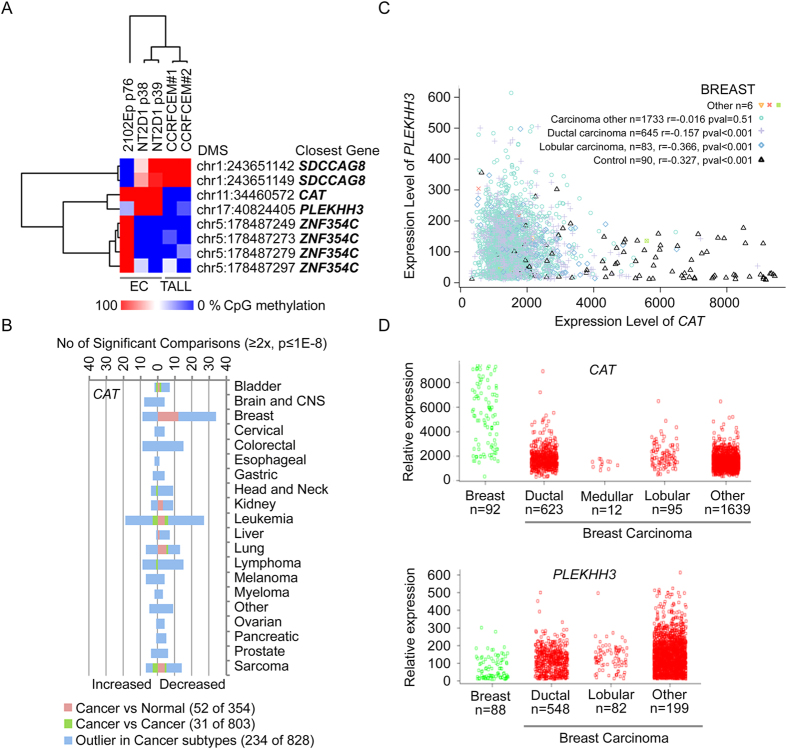
Epigenetic and Transcriptional Regulation of Catalase in Cancers. (**A**) Reduced Representation Bisulfite Sequencing data for the sites differentially methylated in normal and abnormal hPSCs in nullipotent (2102Ep) and pluripotent (NT2D1) embryonal carcinoma (EC) and T cell acute lymphoblastic leukemia (CCRFCEM, TALL). (**B**) Differential expression (fold change ≥ ± 2.0, p ≤ 1-E8) of *CAT* gene in cancer comparisons (Oncomine database, total 715 studies, 86 733 samples). (**C**,**D**) Relative expression and correlation of *PLEKHH3* and *CAT* in healthy breast and breast cancer samples (IST Medisapiens database). See Table SII.

Next we examined regulation of *CAT* gene in cancers. For this purpose we included in the analysis the pluripotent NT2D1 embryonal carcinoma cancer stem cell line and CCRF-CEM T cell acute lymphoblastic leukemia line, as we have previously shown that growth of these cancer lines are, similarly to karyotypically abnormal hPSCs, sensitive to histone deacetylase inhibition. Furthermore, in abnormal hPSCs several genes associated with acute T cell lymphoblastic leukemia respond to histone deacetylase inhibition whereas in normal cells they do not indicating putative common epigenetic mechanism regulating growth of transformed cells^4^. Similarly to abnormal hPSCs, our RRBS data revealed hypermethylation of the CpG site (Fig. 4A, Table SII) and the surrounding CpG sites (Fig. S2) in *CAT* gene in pluripotent (NT2D1) and nullipotent (2102Ep) embryonal carcinoma cells (EC), whereas in T cell acute lymphoblastic leukemia (CCRF-CEM) the sites were hypomethylated. Examination of cancer associated transcriptome data throughout the collection of 86 733 samples in the Oncomine database (www.oncomine.org, 2015, Thermo Fisher Scientific, Ann Arbor, MI) revealed decreased expression of *CAT* in several cancer types, including sarcoma, leukemia, bladder, breast, kidney, liver and lung cancer (fold change ≥2, p ≤ 1E-8) when compared to healthy corresponding tissues (Fig. 4B, Table SII). In addition, differential expression of *CAT* was observed between several different cancer subtypes and in outlier analysis. As an example, breast carcinoma was among the top cancers showing frequent repression of CAT gene expression. This finding was further supported by the data available IST Medisapiens database (ist.medisapiens.com). Although, in contrast to *CAT*, *PLEKHH3* was hypomethylated in abnormal hPSCs and did not show clear transcriptional changes in stem cells, in healthy and cancerous breast tissues we observed inversed correlation of *CAT* and *PLEKHH3* expression (Fig. 4C,D). This indicates that the epigenetic alterations associated with genomic instability of hPSCs have also relevance in regulation of genomic stability in somatic tissues. However, interpretation of the cancer data is challenging as, in addition of complexity of cancers, often the appropriate healthy reference samples are lacking or are unknown.

Genomic alterations in hPSCs have similarities with those observed during embryonal carcinoma progression^12^. Consistently, our findings indicated that similarly to abnormal hPSCs also embryonal carcinoma cells have hypermethylated *CAT* promoter (Fig. 4A) and do not express CAT protein (Fig. 2E). Therefore, we examined whether embryonal carcinoma *in vivo* lack of CAT protein. Tissue immunohistochemistry of healthy testis and different testicular germ cell tumours (TGCTs) revealed that CAT is expressed by Leydig cells as well as by cells with hematopoietic origin in all the examined samples. In contrast, cells of spermatogenic series from adult tissues were negative for CAT. In tumour samples, all tumour cells were primarily negative for CAT immunoreactivity including the malignant cells of embryonic carcinoma (Fig. S3). The lack of CAT in embryonal carcinoma *in vivo* was consistent with our observations on abnormal hPSCs and embryonal carcinoma cells maintained *in vitro*. However, as our data lacks the CAT levels in fetal germ cells, which give rise to embryonal carcinomas, it remains still to be further elucidated whether silencing of CAT may have relevance in the development of germ cell tumors. However, as shown in Fig. 4B, altered *CAT* expression can be observed in numerous cancer types. Whether the regulation is epigenetic and whether CAT has a key role in tumorigenesis remains to be further elucidated.

Our results highlight that abnormal stem cells carry common epigenetic defects in their genome affecting transcription of genes regulating pluripotency, cell adhesion, developmental processes and in particular response to oxidative stress. Importantly, these epigenetic alterations correlate with aberrant regulation in cancers. In particular our results highlight potential importance of *CAT* in the regulation of genomic integrity of hPSCs. Catalase is a key antioxidant enzyme with a conserved and crucial function in defence against aging, oxidative stress, toxic compounds and DNA damage^13^,^14^,^15^. Interestingly, we observed abiotic generation of H_2_O_2_ in the hESC culture medium exposing hESCs in these conditions to increase levels of oxidative stress. However, although according to our results karyotypically abnormal cells lack the CAT protein, the capacity of karyotypically normal and abnormal hESCs to eliminate H_2_O_2_ was similar, indicating that CAT is not needed for this function in hESCs. Nevertheless, as silencing of *CAT* was the strongest consistent alteration associated with genomic instability of hESCs, we believe this enzyme must have an important function in protecting genomic stability of hESCs through mechanisms that remain to be further elucidated. Our findings provide novel insights into the regulation of genomic and epigenomic stability of hPSCs during spontaneous transformation towards malignant fate. Furthermore, monitoring of the DNA methylation status of *CAT* in hPSCs may provide valuable tool for estimating quality and sensitivity of the cells to genomic insults. Further in depth studies are needed to address this and other remaining questions, such as what mechanisms are important in regulating the oxidative control in hPSCs, what is the functional significance of CAT in hPSCs and whether CAT is essential for the maintenance of genomic integrity of human stem cells at different developmental stages.

## Methods

### Cell cultures

The pluripotent human embryonic stem cell lines (HS360, H9) maintained in Turku, Finland were expanded on mitotically inactivated human foreskin fibroblasts and before experiments maintained in feeder free conditions on Matrigel and in mTeSR1 at least for two passages. The H7, H14 and Shef5 cells in Sheffield, UK were maintained on mouse feeders in standard FGF2 containing hESC medium as previously described^4^. In both laboratories the cells were grown in tissue culture incubators at 37 °C and 5% CO2. The karyotypes (Table SIII) of the lines were determined with either G-banding or Karyolite BoBs method^16^. Some of the H7 samples (H7.Np30, H7.Np38, H7.ABp128, H7.ABp132, H7.ABp230, H7.ABp237) have been previously genotyped with Affymetrix SNP6 arrays^17^.

### Reduced Representation Bisulfite Sequencing

Genomic DNA was extracted from the cells and processed for Reduced Representation Bisulfite Sequencing as previously described^7^,^8^. The libraries were sequenced with Illumina HiSeq2000 platform or Illumina HiSeq2500 platform using 1 × 50 bp chemistry. The total number of reads obtained from sequencing was 9.8M–24.4M per sample. Raw data available through http://www.ncbi.nlm.nih.gov/bioproject with accession code: PRJNA310646. Poor quality bases and adapter containing bases were trimmed off using Trim galore with custom parameters. Only reads with at least 20 bp were included in the downstream analysis. After quality trimming 96–99.26% of total reads (8.6M–24.7M) were left. Quality processed reads were mapped to human genome (hg19) using Bismark^18^. Mapping efficiency for the hg19 genome was 56.6%–73.9% resulting in 5.5M–14.04M mapped reads. Bisulfite conversion efficiency was estimated using the lambda genome and it was found to be above 99.5% for all the samples, except h7_s6_p222 which was 98.99%. Hg19 mapped reads were processed with Bismark methylation caller to estimate the methylation status of each cytosine in every mapped read. Only the CpGs with at least 5x coverage (945K–1.5M CpGs) were included in the further analysis. Clustering (Fig. S4) and differential methylation analysis was performed using methylKit^19^. The CpGs with at least 25% difference in the methylation status with the qvalue cut-off 0.05, and pvalue adjusted to qvalue, were considered to be differentially methylated. The sex chromosomes X and Y were excluded from the analysis. The genes within closest distance to the differentially methylated sites were retrieved with GREAT Annotation Tool. Ingenuity Pathway Analysis Tool (Qiagen) was utilized for exploring functions and networks of the genes with closest distance to the altered sites^20^. GENE-E visualization platform (www.broadinstitute.org/cancer/software/GENE-E/index.html) was used to generate heatmaps.

### RNA sequencing

Normal and abnormal H9 cells and HS360 cells were analysed with mRNA-seq. The RNA was extracted simultaneously from the same cells as DNA with Qiagen Allprep kit. The Next-Generation Sequencing libraries were prepared with Illumina TruSeq mRNA-seq kit according to the protocol from the manufacturer. The libraries were sequenced with 1 × 50 bp chemistry with Illumina HiSeq2000 platform. The total number of reads obtained from sequencing was 19M–28M per sample. The data was analysed in Illumina’s BaseSpace cloud (https://basespace.illlumina.com) with RNA Express v1.0, which applies STAR aligner for read alignment^21^. Of the total reads 97.51–98.6% were aligned to hg19 reference genome. The differential gene expression analysis, with cut-offs indicated in the results, was carried out in with RNA Express v1.0 and DESeq2^21^,^22^. Ingenuity Pathway Analysis Tool (Qiagen) was utilized for exploring functions and networks of the altered genes.

### Western blot

Cells were processed for western blot analysis as previously described^4^. Lysates were electrophoresed on a 4–20% SDS-PAGE gel (Criterion TGX precast Gel, Bio-Rad) and transferred to a nitrocellulose membrane. Membranes were incubated overnight at +4 °C with primary antibodies: Catalase (Santa-cruz, sc-50508, 1:250) Ku70 (Abcam, ab2620 1:5000), Signal was detected with Pierce developing solution (Pierce, Thermo Fisher Scientific).

### Reactive oxygen species assay

Human H9 ESC lines with normal and abnormal karyotype (trisomy of chromosome 12) were harvested with Accutase, counted and plated on Matrigel-coated 96-well plates in mTeSR1 (Stem Cell Technologies). ROS assay (ROS-Glo™ H2O2, Promega) was carried out to according to the kit manual following the Cell-Based assay. Also matrigel-coated wells with mTeSR1 and without cells were included in the measurements. The luminescent signal was measured 2–3 hours after incubation with H_2_O_2_ substrate and after 20 min incubation with ROS-Glo^TM^ reagent with Luminoskan Microplate Luminometer (Thermo Scientific). Three biological and four biological replicates were carried out for karyotypically normal and abnormal cells, respectively, and the signals for each replicate were measured from at least 9 wells.

### Tissue specimens

Human testicular tissue samples representing healthy testis, benign teratoma, embryonal carcinoma and two mixed germ cell tumors were used to assess catalase production *in vivo* in normal and abnormal situations. Human liver tissue was used as a positive control for catalase activity based on the reported expression of catalase by hepatocytes (see The Human Protein Atlas (http://www.proteinatlas.org/). All samples were obtained from Auria Biobank, Turku, Finland. The samples were fixed in 10% neutral-buffered formalin, embedded in paraffin and cut into 5 μm consecutive sections. The sections were used for hematoxylin and eosin (HE) staining and immunohistochemistry (IHC) analyses. The local ethical committee gave the approval for using the tissue samples.

### Immunohistochemistry of the tumour tissues

Immunohistochemistry for catalase (CAT) was performed accordingly to protocols previously described^23^ with minor modifications. For antigen retrieval deparaffinized and rehydrated tissue sections were digested with 0.01% trypsin (Sigma) in PBS for 5 min in +37 °C followed by microwaving in 10 mM Na-citrate buffer at pH 6.0 for 15 min at 700W in a conventional household microwave (Elextrolux). Then, the sections were cooled for 20 min without changing the buffer. A primary rabbit polyclonal antibody to human catalase (H-300, dilution 1:50, Santa Cruz Biotecnology Inc, Santa Cruz, CA) was used for CAT IHC. Immunohistochemical stainings for low-molecular-weight keratins (CXPan), placental alkaline phosphatase (PLAP) and CD30 were performed with ready-to-use antibodies with Benchmark XT immunostainer and ultraView Universal DAB Detection Kit (Ventana/Roche, Tucson, Arizona, USA).

## Additional Information

**How to cite this article**: Konki, M. *et al.* Epigenetic Silencing of the Key Antioxidant Enzyme Catalase in Karyotypically Abnormal Human Pluripotent Stem Cells. *Sci. Rep.* **6**, 22190; doi: 10.1038/srep22190 (2016).

## Supplementary Material

Supplementary Information

Supplementary Dataset 1

Supplementary Dataset 2

Supplementary Dataset 3
