# Supplementary material for: Epigenetic Silencing of the Key Antioxidant Enzyme Catalase in Karyotypically Abnormal Human Pluripotent Stem Cells
Source: Sci Rep. 2016 Feb 25;6:22190. doi: 10.1038/srep22190 (PMC4766493; doi:10.1038/srep22190)
Supplement: Supplementary Information [file srep22190-s1.docx]

**SUPPLEMENTAL FIGURES**

**Epigenetic Silencing of the Key Antioxidant Enzyme Catalase in Karyotypically Abnormal Human Pluripotent Stem Cells**

Mikko Konki^1*^, Kalyan Pasumarthy^1*^, Maia Malonzo^2*^, Annele Sainio^3^, Cristina Valensisi^1, 4^, Mirva Söderström^5^, Maheswara Reddy Emani^1^, Aki Stubb^1^, Elisa Närvä^1^, Bishwa Ghimire^1^, Asta Laiho^1^, Hannu Järveläinen^3^, Riitta Lahesmaa^1^, Harri Lähdesmäki^2^, R. David Hawkins^1, 4^, Riikka J. Lund^1,6^

**Supplemental Figures**

**
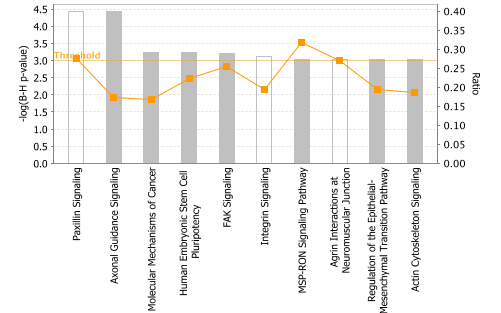
**

**Figure S1. The Main Functional Categories of the Genes with Altered Transcriptional and Epigenetic Regulation in Karyotypically Abnormal Human Pluripotent Stem Cells (H7 and H9 lines).** The genes within closest distance (basal+extension: 5000 bp upstream, 1000 bp downstream, 50 000 bp max extension) from the differentially methylated sites in karyotypically abnormal H7 and H9 lines were retrieved with Great Annotation Tool. The RNA-seq data was integrated to DNA methylome data to identify the genes displaying also altered transcriptional regulation (fold change ≥2, adjusted p≤0.05). Ingenuity Pathway Analysis tool (Qiagen) was used to identify the top functional categories of the affected genes. In the figure the statistical significance (Fisher’s exact test) of the functional enrichment is shown on primary y-axis and indicated by the height of the bars, whereas the ratio on secondary y-axis, represented by dotted orange line, indicates the proportion of altered molecules in each functional category (x-axis).


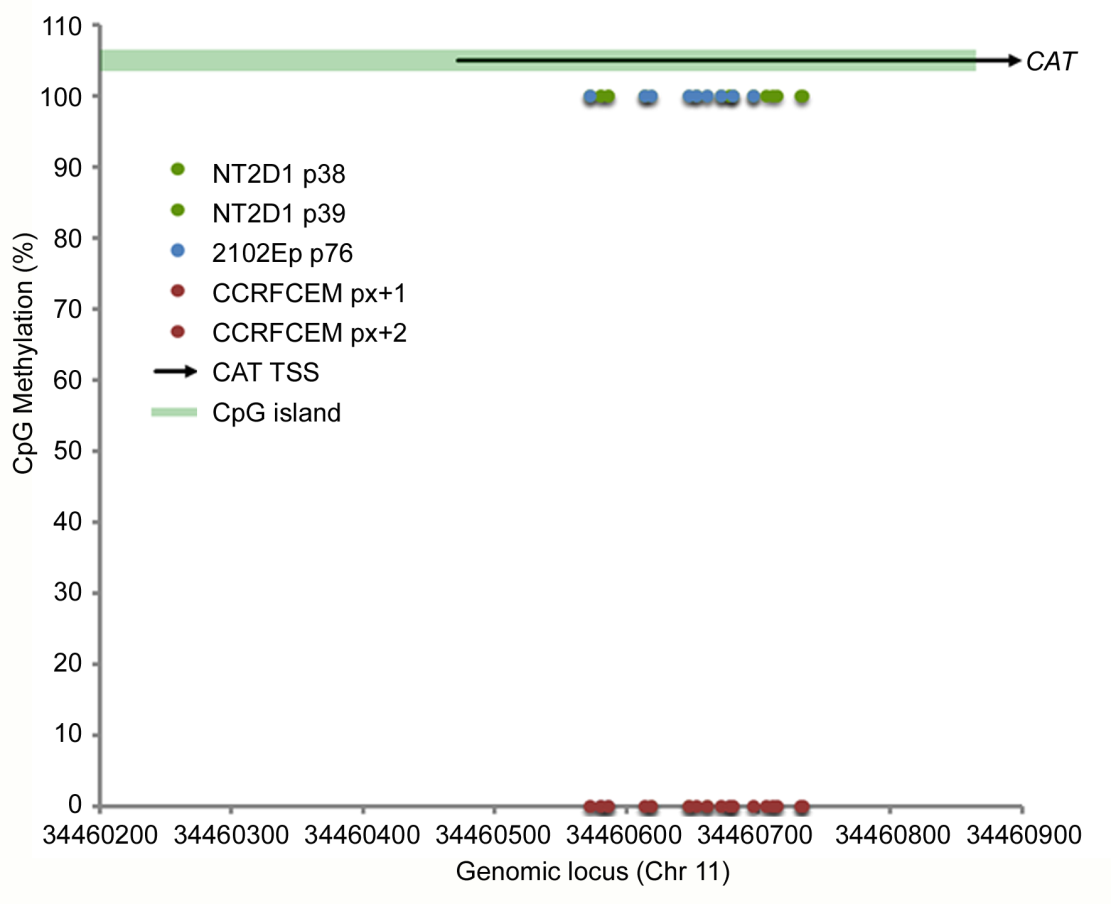


**Figure S2. CpG Methylation Status of Promoter Region of Catalase Gene in Cancer Cell Lines.** CpG methylation levels of the CpG island in Catalase (CAT) gene in pluripotent Embryonal Carcinoma cells NT2D1 (green), nullipotent Embryonal Carcinoma cells 2102Ep (blue) and T Cell Acute Lymphblastic Leukemia CCRFCEM (red).


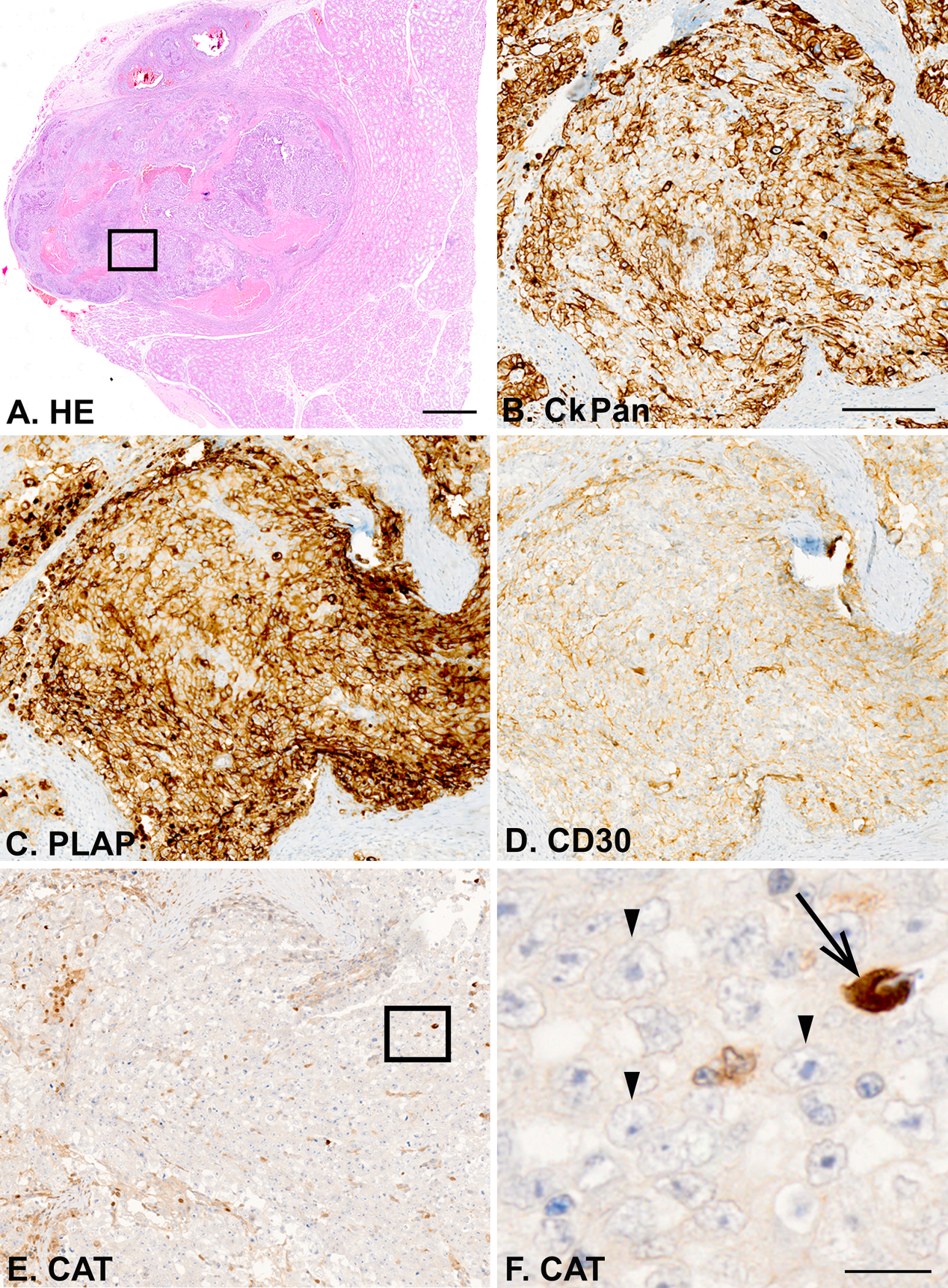


**Figure S3. Embryonal Carcinoma Cells are Negative for Catalase Immunoreactivity.** Embryonic carcinoma is characterized by positivity for CkPan, PLAP and CD30. **A**. HE staining of embryonal carcinoma in the testis. Images B-E are magnified views of the marked area in image A. **B**. IHC for CkPan. **C**. IHC for PLAP. **D**. IHC for CD30. **E**. IHC for catalase. **F**. Magnified view of marked area in image E. Arrow in image F indicates catalase positive lymphocyte and arrowheads catalase negative embryonal carcinoma cells. Scale bars in images A. = 2 mm, B.-E. = 200 µm and F. = 20 µm, respectively.

##
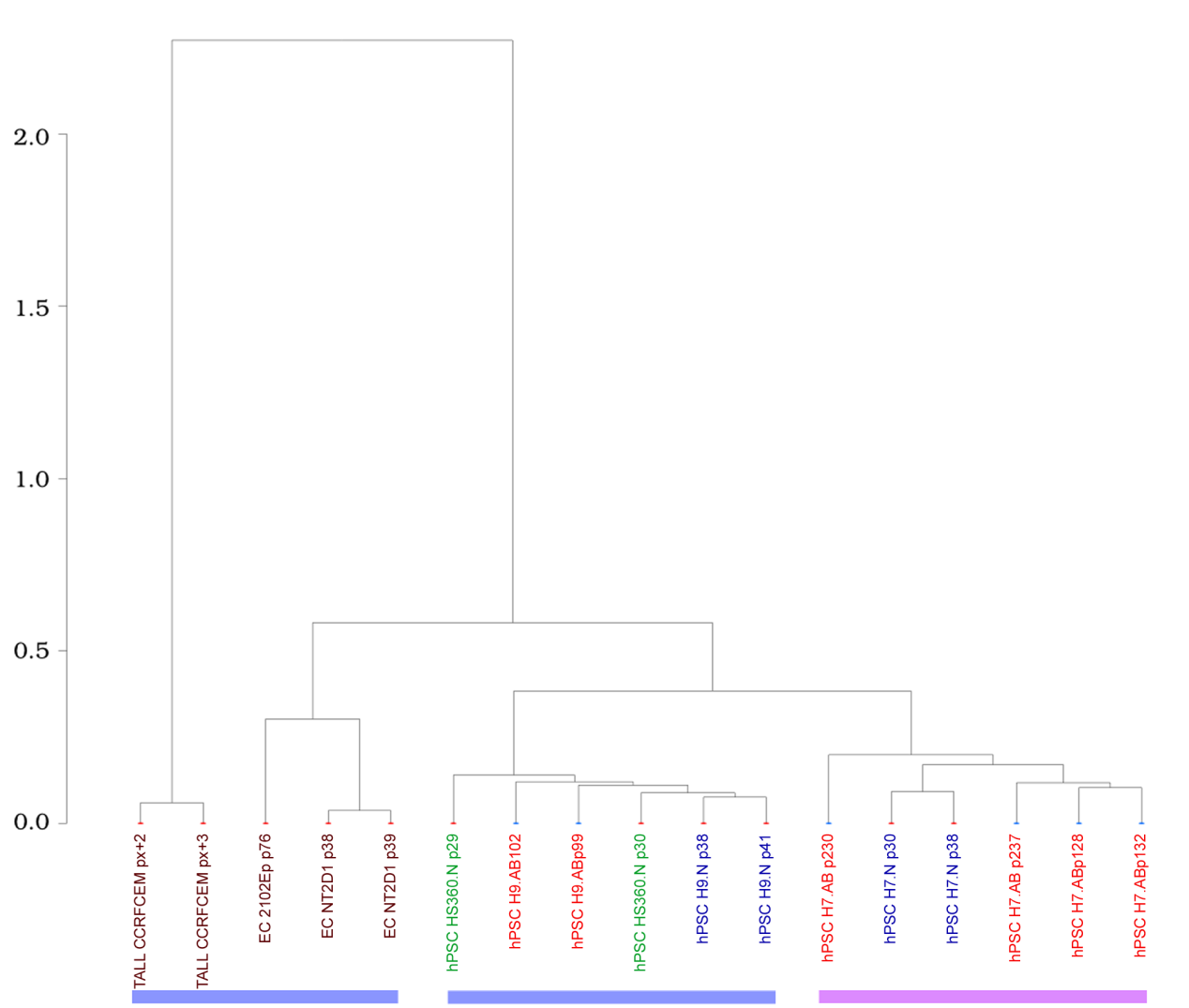


**Figure S4. Clustering of cell lines based on their CpG methylomes.** Clustering analysis (distance method = correlation, clustering method = ward) was carried out for the DNA methylomes (5x coverage) of the indicated cell lines. In the figure are the Wisconsin H7 (maintained in Sheffield, UK) and H9 (maintained in Turku, Finland) hPSC lines before (N=normal) and after (AB=abnormal) spontaneous transformation to abnormal karyotype, normal Swedish HS360 hPSC line (maintained in Finland), which does not tend to accumulate karyotypic abnormalities, nullipotent (2102Ep) and pluripotent (NT2D1) embryonal carcinoma cell (EC) and CCRF-CEM acute T cell lymphoblastoma cell (TALL) lines. The color bars indicate the laboratory where the cells were maintained at sampling (blue=Finland, purple=UK).
